# Outcomes of Microvascular Decompression Surgery: A Study on Cerebrospinal Fluid Leakage and Surgical Techniques. Comment on Lee, H.S.; Park, K. Dura Closure Tactics to Prevent CSF Leakage in Microvascular Decompression Surgery. *Life* 2025, *15*, 574

**DOI:** 10.3390/life15071055

**Published:** 2025-07-01

**Authors:** Laiba Saher

**Affiliations:** Karachi Medical and Dental College, Karachi 74700, Pakistan; saherlaiba17@gmail.com

I have critically appraised the article “Dura Closure Tactics to Prevent CSF Leakage in Microvascular Decompression Surgery” by Hyun Seok Lee and Kwan Park [1]. This study offers valuable insights, and the authors’ contribution is greatly appreciated, particularly for their thorough discussion of the dural closure technique aimed at preventing cerebrospinal fluid leakage in microvascular decompression surgery. Notably, it highlights the use of a triple-layered closing technique (three-step suturing technique). However, the inclusion of additional elements could further strengthen the study’s conclusions and its clinical relevance.

The authors addressed the most crucial complication, specifically cerebrospinal fluid leakage, commonly witnessed following microvascular decompression surgery. Cerebrospinal fluid (CSF), an ultrafiltrate of plasma, is found in the brain’s ventricles as well as the subarachnoid spaces of the skull and spine. It performs essential tasks, including feeding the brain, eliminating waste, and providing protection [2]. This study determined that the three-layered technique is effective in minimizing cerebrospinal fluid leakage, contrasting with existing research, which examined a single epidural sealing approach (two layers) and a sandwich technique (three layers). The result showed a 7.3% leakage in the sandwich approach and a 10% leakage in the single-layer technique, with no discernible difference in CSF leak rates [3].

The lack of a control group in the analysis of traditionally used two-layered closure techniques makes it challenging to compare the methods and determine the more effective approach. In contrast to a previous study, which included a control group and evaluated two-layer approaches, this paper suggests that these techniques might not offer the same degree of watertight closure. This study demonstrated that a three-layer reconstruction employing fleece-bound tissue sealing effectively prevented CSF leakage, achieving a 98% success rate. This supports the idea that the three-layer closure is more reliable than the two-layer technique [4].

Moreover, the use of a retrospective design instead of randomization and blinding introduces potential inherent biases, including incomplete data and selection errors. Most importantly, there was a lack of long-term follow-up, limiting the study to short-term outcomes. The subjects were only evaluated during their hospital stay, thereby preventing the assessment of any delayed cerebrospinal fluid leakage. This does not ensure whether the technique holds up over months and longer. Furthermore, this is a single-surgeon, single-center study, which affects its validation, as outcomes can vary with experience and technique. A multi-center study is recommended for stronger generalization. 

Furthermore, although the triple-layer technique discussed is often effective, it might increase operative costs. However, the economic analysis is not addressed. Mastoid air cells are briefly mentioned, but various strategies for sealing them are not presented. Moreover, the researchers used simple materials with known efficacy, commonly used in neurosurgery, strengthening the validity of the study. The impact of race and ethnicity on study outcomes were not explored.

According to the study, the closure materials utilized did not result in any postoperative infections, enhancing the triple-layer technique’s safety profile. Additionally, postoperative patient outcomes like pain, patient satisfaction, and quality of life should be analyzed following surgery. The diagnosis of cerebrospinal fluid leak rhinorrhea and the mechanistic features of triple layering have neither been standardized nor emphasized. In addition, the preoperative validation and results of MRI and CT scan could have been interpreted more thoroughly for a better understanding.

The article is very informative but lacks comparative evidence. Future randomized control trials are necessary, and control groups must be included to assess the effectiveness of the technique. Patients’ outcomes need to be evaluated, multi-center studies with different surgeons will be essential to account for variability. These gaps must be addressed to draw more reliable conclusions based on solid evidence.
